# Identification and monitoring of brain activity based on stochastic relevance analysis of short–time EEG rhythms

**DOI:** 10.1186/1475-925X-13-123

**Published:** 2014-08-28

**Authors:** Leonardo Duque-Muñoz, Jairo Jose Espinosa-Oviedo, Cesar German Castellanos-Dominguez

**Affiliations:** Grupo de Automática y Electrónica, Instituto Tecnológico Metropolitano, Medellin, Colombia; Grupo GAUNAL, Universidad Nacional de Colombia, Medellin, Colombia; Signal processing and Recognition Group, Universidad Nacional de Colombia, Manizales, Colombia

**Keywords:** Stochastic relevance, EEG rhythms, Interictal/ictal classification, Epilepsy monitoring

## Abstract

**Background:**

The extraction of physiological rhythms from electroencephalography (EEG) data and their automated analyses are extensively studied in clinical monitoring, to find traces of interictal/ictal states of epilepsy.

**Methods:**

Because brain wave rhythms in normal and interictal/ictal events, differently influence neuronal activity, our proposed methodology measures the contribution of each rhythm. These contributions are measured in terms of their stochastic variability and are extracted from a Short Time Fourier Transform to highlight the non–stationary behavior of the EEG data. Then, we performed a variability–based relevance analysis by handling the multivariate short–time rhythm representation within a subspace framework. This maximizes the usability of the input information and preserves only the data that contribute to the brain activity classification. For neural activity monitoring, we also developed a new relevance rhythm diagram that qualitatively evaluates the rhythm variability throughout long time periods in order to distinguish events with different neuronal activities.

**Results:**

Evaluations were carried out over two EEG datasets, one of which was recorded in a noise–filled environment. The method was evaluated for three different classification problems, each of which addressed a different interpretation of a medical problem. We perform a blinded study of 40 patients using the support–vector machine classifier cross–validation scheme. The obtained results show that the developed relevance analysis was capable of accurately differentiating normal, ictal and interictal activities.

**Conclusions:**

The proposed approach provides the reliable identification of traces of interictal/ictal states of epilepsy. The introduced relevance rhythm diagrams of physiological rhythms provides effective means of monitoring epileptic seizures; additionally, these diagrams are easily implemented and provide simple clinical interpretation. The developed variability–based relevance analysis can be translated to other monitoring applications involving time–variant biomedical data.

## Introduction

Epilepsy is a chronic neurological disorder characterized by recurrent unprovoked seizures resulting from several brief and episodic neuronal hypersynchronous discharges with dramatically increased amplitudes affecting normal (i.e., background) brain activity. In the clinical setting, however, seizures without overt convulsions and the low probability of observing a seizure during standard recording times of 20-40*min* greatly complicate diagnoses, as noted in [1]. Moreover, due to the excessive presence of artifacts, interference or overlapping symptomatology with other neurological disorders, the discrimination between normal brain activities and epileptiform activities for epilepsy diagnoses can be challenging, even from the visual inspection of an EEG by an experienced neurologist. Even the most highly trained neurology experts are not able to differentiate interictal EEG signals of epileptic from normal EEG data with over 80*%* accuracy. The complete review of recorded EEG signals by a trained professional is time–consuming, as explained in [2, 3]. Therefore, in clinical monitoring, automated EEG data techniques show promise in finding traces of interictal (between seizures) and ictal (during an epileptic seizure) states of epilepsy.

Generally, EEG data reflecting the electrical activity of different brain neuronal dynamics can be described as a collection of several sub–band waveform frequencies (or *physiological rhythms*) ranging between slow to fast activity (***δ***, ***θ***, ***α***, and ***β***). For rhythm analyses, most of the known EEG–based detection systems require features extracted from time, frequency, or time–frequency domains to feed into a given classifier model [4]. Therefore, for epileptic seizure detection, features extracted in either time or frequency are assumed to have less computational complexity and burden [5]. However, due to the non–stationary behavior of EEG recordings, time–frequency domain methods typically lead to higher successes [6–9]. Moreover, it is known that the time–frequency representation ability to analyze different neural rhythm scales can be used as a reliable EEG marker; this ability has been shown to be a powerful tool for investigating small–scale neural brain oscillations [10, 11]. Accordingly, close relationships are often established between rhythms and epileptic seizures because they highly vary with changes in a nonictal/ictal state [12]. Particularly, the ***δ*** and ***θ*** rhythms that exhibit lower frequencies and higher magnitudes with respect to ***α*** waves may occur in epilepsy cases. Consequently, a quantitative contribution of each frequency sub–band must be clearly expressed toward automatic epileptic seizure identification and monitoring.

In order to measure the contribution of time–variant rhythms to the representation of brain activity, the following two stages must be carried out: *i*) the estimation of physiological rhythms highlighting the non–stationary behavior of EEG data, and *ii*) the construction of a measure appraising the concrete amount, or relevance, of each extracted rhythm dynamics in terms of discriminating different brain neuronal activities. In the former stage, several time–frequency decompositions have been proposed to encode EEG dynamics in extracted rhythms. These enhancing methods range from the recently introduced Empirical Mode Decomposition (EMD) [13–15] and the baseline Short Time Fourier Transform (STFT) [5, 6, 16] (including the use of several time–frequency distributions [7, 17]), to the Wavelet Transform (WT), which seems to be the most commonly used decomposition [1, 3, 5, 9, 16, 18–22].

In the latter stage, the knowledge of individual latent time–variant components has been proven to supply useful insight into EEG data analysis. In particular, as discussed in [1, 3, 7, 19, 23, 24], principal component analysis (PCA) has been used in feature enhancement for epilepsy classification. Additionally, to determine all clinically relevant EEG waveforms, independent component analyses [25] and more elaborate manifold learning techniques have been also applied [26]. Overall, it was found that based on a time–frequency EEG decomposition, one may measure the relevance of each frequency sub–band by an analysis or classification performance. However, during the relevance evaluation stage, none of these techniques take into account the non–stationary behavior of the epileptic–related rhythmic activity [7, 11, 27]. Therefore, to improve the classification accuracy, the identification of brain neuronal activities should be based on the stochastic relevance analysis of short–time EEG rhythms.

We aim to develop a methodology based on the stochastic relevance analysis that estimates the contribution of each time–variant EEG rhythm to discriminate between normal and ictal/interictal states. For this purpose, a subspace–based stochastic analysis of EEG rhythm dynamics is introduced. Thus, instead of a widely used scalar–valued parameter set extracted from a given EEG signal, neuronal states are detected throughout this analysis by using a vector set of short–time rhythms. The Short Time Fourier Transform is used as an enhancing decomposition to provide suitable temporal and spectral resolutions of extracted EEG rhythms [7, 28]. To show the robustness of the proposed training approach, the developed methodology is tested over two EEG datasets, one of which is recorded in a noisy (i.e., non–shielded) environment. We perform a blind study of 50 patients using the support-vector-machine classifier cross–validation scheme under three epilepsy–related problems of medical interest. As a result, our methodology provides support to the identification of brain neural activity related to epileptic seizure diagnoses. Furthermore, we introduce the concept of the relevance rhythm diagram, which provides a simple clinical interpretability that is implementable in automated EEG monitoring systems. This paper is organized as follows: in section “Materials and methods”, we briefly introduce the estimation of short–time EEG rhythms and their stochastic relevance evaluations. In section “Experimental setup”, an experimental set–up illustrates the effectiveness of the proposed training approach. Finally, the discussion and conclusions of obtained results are given in sections “Discussion” and “Conclusions”, respectively.

## Materials and methods

### Short–time rhythm extraction from enhanced EEG representation

The main goal of a time–frequency representation is to decompose a given EEG signal into its time variant spectral components, thereby properly encoding non–stationary dynamics. In particular, based on a Fourier Transform, the STFT introduces time localization by using a sliding window function, *ϕ*(*t*), with an EEG signal, *y*(*t*). The spectral density of EEG data can be calculated by means of a *spectrogram*[7]:
1

The rhythms carrying out clinical and physiological interest fall primarily within the following four frequency sub–bands: *Delta* indicated as ***δ*** with frequencies *f* < 4 *Hz*), *Theta* (***θ***, *f* ∈ [ 4,8] *Hz*), *Alpha* (***α***, *f* ∈ [ 8,13] *Hz*), and *Beta* rhythms, (***β***, *f* ∈ [ 14,30] *Hz*). A short–time version of EEG rhythms, ***x***_*m*_ ∈ {***δ***,***θ***,***α***,***β***}, *m*=1,…,*p*, can be extracted from the discretized spectrogram, *S*_*y*_(*l*,*k*), by using several spectral envelope representations. Because the known discrete cepstrum is more suitable for the STFT representation of nearly periodic signals, the time–variant EEG rhythms are explicitly extracted using the *Frequency Cepstral Coefficients* subseries, ***z***_*n*_ = {*z*_*n*_(*l*): ∀*l* ∈ *T*}, and computed through the Discrete Cosine Transform of triangular log–filter banks, {*F*_*m*_(*k*) : *m*=1,…,*p*}, that are spaced in the frequency domain as Eq. 2 [28]:
2

where *n*_*M*_ is the desired Cepstral coefficient feature set, and *s*_*m*_(*l*) is the weighted sum of each frequency filter response set, defined as . Variables *m*, *l* and *k* are indices for filter ordinal, time, and frequency axes, respectively; *n*_*K*_ is the number of samples in the frequency domain. Therefore, each Cepstral coefficient subseries is associated directly with one of four EEG rhythms, that is, ***z***_*i*_→***x***_*i*_: ∀*i*=*i*,…,4.

We select the linear filterbank LFCC for representation of EEG signals because they may more accurately refined to each rhythm frequency bandwidth. Therefore, we use five cepstral coefficients associated with *δ*, *θ*, *α*, and *β* rhythms, extracted as dynamic features(which were also used for EEG analysis in [29, 30]).

As a result, instead of a widely used scalar–valued parameter set extracted from the EEG signal, neural activities relating to epileptic seizures are detected by using a vector set of short–time rhythms,  with *t* ∈ *T*, which carries temporal information on non–stationary EEG recordings. The variable *p* represents each rhythm, i.e., ***δ***,***θ***,***α***,***β*** waveforms are denoted as *i*=1,2,3,4, respectively. Therefore, the input stochastic feature set, {***x***_*i*_}, is represented by the observation ensemble comprising *M* objects in the following input observation matrix, ***X***=[*X*_1_|⋯|*X*_*m*_|⋯|*X*_*M*_]. In turn, each object, denoted as *X*_*m*_, *m*=1,…,*M*, is described by their respective observation set of short–time vectors, , such that each column becomes  for  where each vector,  is a measured EEG rhythm (equally sampled through time), and is  the *i*–th stochastic waveform of the *m*–th object for a given time *t* instant.

### Stochastic relevance analysis of short–time rhythms

In order to analyze the ability of the rhythm discriminant to detect neural brain states, priority is placed on identifying the time evolution and the structure of the underlying short–time waveforms, i.e., their contribution over time to the classifier performance must be carefully analyzed and quantified [5]. In this regard, the relevance analysis of the time–variant signals is carried out.

Aiming to take into consideration the non–stationary behavior of short–time rhythms, this work discusses the use of subspace–based analysis when the stochastic feature set is written as a linear combination of *q*<*p* independent basis functions. Namely, the minimum mean square–based error is assumed as the evaluation measure of the linear transformation on subspaces, . Thus, the set of orthogonal vectors is estimated. The resulting minimum weighted linear combination, *q*, can approximate ***x***, in such a way that data information is maximally preserved. That is,
3

where  is the reconstruction of ***x***_*i*_, ∥·∥_2_ is the norm squared value, and the notation ***E***{·} represents the expectation operator. We assume ***x*** to be a zero mean, random *p* dimensional vector with a covariance matrix  which is approximated by a simpler vector, , in the form:  where ***A*** is some matrix of dimension  such that *q* <*p*. The requirement in Eq. (3) to preserve maximum data information is rewritten as follows:
4

The solution of Eq. (4) implies  where  is the eigenvector set of the diagonal covariance matrix ***Σ***_***X***_ with non–ranked singular values {*λ*_*k*_ : *k* ∈ *p*}. In practice, the covariance matrix is estimated as  with a size of *pT* × *pT*. In most cases, *pT* ≫ *M*; we cannot readily compute eigenvectors and eigenvalues of such a large matrix. However, the needed eigenvector set is computed, based on the rank property stating that  where  the eigenvector set of the matrix ***X***^⊤^***X*** with the same non–null eigenvalues as ***XX***^⊤^.

Magnitudes of eigenvector entries spanning the representation basis are then chosen as shown by relevant measurements in [31]. Namely, for a value of *q* chosen by the explained variance criterion  where *τ* = 1,…,*pT* and  is the set of singular values ranked in decreasing amplitudes. Therefore, the measured contribution of each *i* EEG rhythm is given by ***g***_*i*_(*t*) =[ *g*((*i*-1)*T*+1) …*g*((*i*-*t*)*T*+1) …*g*(*iT*)]. However, with the provided linear transformation in Eq. (3), a piecewise stationary restriction should also be imposed, any estimator of feature relevance ***g***_*i*_(*t*) should remain constant within a given time frame *Δt* ∈[ (*i*-1)*T*+1,*iT*]. That is,
5

For identifying EEG rhythms that have the most significant influence, and thereby provide, an estimation of relevance measures for each dynamic, the standard average is assumed as follows [32]:
6

The numerical evaluation of Eq. (6) yields the *relevance weight* of the *i*–th stochastic feature. The main assumption of the proposed relevance analysis is that the larger the weight, the more relevant the stochastic feature. Consequently, the set of estimated weights {*γ*_*i*_} can be ordered by decreasing values of achieved relevance, i.e.,  where  for  and 

### Support vector machine classifier

Given a short–time training data set {*X*_*m*_,*ξ*_*m*_:*m*∈*M*} composed of *M* stochastic objects, where input  and output *ξ*_*m*_ ∈ ±1, the SVM searches for a hyperplane ***w***^⊤^*X* + *b*= 0, as a boundary separating positive and negative values from each other at maximum margins. Here, ***w*** is the hyperplane normal and *b* is a bias. In the bi–class task case, the optimum boundary chosen under the maximal margin criterion is found by minimizing the function, *J*, as follows [31]:
78

where  is a tradeoff penalization parameter indicating the relative importance of the model complexity when compared with the training error, and  is the training error for the *m*–th sample.

The above optimization problem can be solved by several quadratic methods resulting in the optimum decision boundary in the form of a linear combination of the termed support vectors, that is,  where *α*_*m*_≠0. To extend the solution to nonlinear boundary problems, functions expressing the dot product of two vectors on input space (i.e. *Kernels*) can be introduced. We use the Radial Basis Function (RBF) kernel with a dispersion parameter, *ς*, and the classifier tradeoff parameter, *c*.
9

Both parameters are adjusted using a particle swarm meta-heuristic optimization, a bio-inspired method used for SVM parameter determination, as carried out in [33]. In practice, the use of a Gaussian kernel is more desirable than Laplacian or Polynomial kernels because it creates a Reproducing Kernel Hilbert Space with universal approximating capabilities, as discussed in [34]. Polynomial kernels are less widely used than the RBF kernel. For similar training and testing costs, a polynomial kernel may not provide greater accuracy than the RBF kernel, as suggested in [35].

## Experimental setup

**Figure 1:**
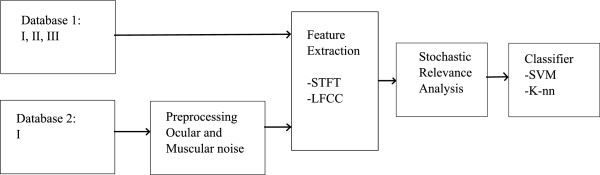
**Training scheme of the proposed methodology of brain neural activity identification, based on stochastic relevance analysis of short–time EEG rhythms.**

Based on the stochastic relevance analysis of short-time EEG rhythms, the proposed methodology of brain neural activity identification comprises the next stages, as shown in Figure 1: *a*) preprocessing, *b*) a short–time rhythm estimation from the STFT spectrogram, *c*) a stochastic analysis and the computation of the rhythm relevance weights, and *d*) an estimation of the classifier performance based on estimated relevance weights.

### Electroencephalographic recording datasets

In order to validate the proposed methodology of brain neural activity identification, this work uses the following two EEG data sources. The signals were obtained retrospectively during medical examinations, performed in accordance with the Declaration of Helsinki. The data were anonymized and stored in the output format (txt files). Database one was acquired in Klinik für Epileptology, Universitat Bonn, Germany. Database two was acquired under approbation of the Ethics Committee of the Instituto de Epilepsia y Parkinson del Eje Cafetero (Pereira, Colombia).

#### Database One (DB1):

This collection is publicly available. The complete data set consists of five subsets [22, 36] (A, B, C, D, and E). Each set is composed of 100 single–channel EEG segments of 23.6*s* duration. Sets A and B are taken from five healthy subjects with eyes opened and closed, respectively. All signals from sets C, D and E come from five epileptic subjects. Sets C and D comprise seizure–free interictal signals measured on the epileptic zone and on the hemisphere opposite to the hippocampal formation of the brain. Set E contains epileptic signals recorded from each aforementioned location during an ictal seizure. Sets C, D and E were recorded intracranially.

All EEG signals were digitized at 173.61 *Hz* and 12– *bit* resolution. To retain relevant EEG data signals were filtered through a low–pass filter with a 40 *Hz* cutoff frequency.

The data set DB1 was used on three problems, which are of medical interest and have been widely studied in literature [7]:

- *Bi–class Problem I,* for which normal (A-type) and seizure (E-type) labeled recordings are distinguished.
- *Problem II,* or a three–class problem, closely represents real medical applications, including three categories: normal (A-type EEG segments), seizure–free interictal (D-type EEG segments), and seizure (E-type EEG segments).
- *Problem III*, or a five–class problem, i.e., all five classes are investigated, wherein all EEG segment sets from the above described dataset is used: normal (Types A and B), interictal (Types C and D) and seizure (Type E).

#### Database Two (DB2):

**Figure 2:**
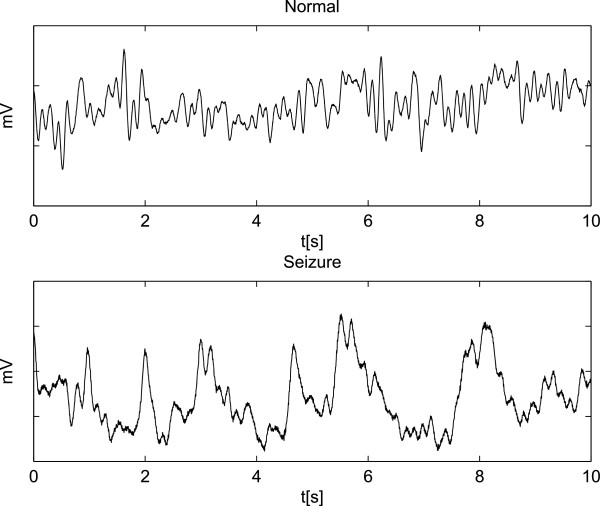
**EEG recorded in a non-shielded environment.** Examples of 10-*s* segments for normal (A) and epileptic seizure (E) activities, taken from the contaminated database DB2.

This bi-class collection, which belongs to the *Instituto de Epilepsia y Parkinson del Eje Cafetero*, contains two subsets of Problem I (i.e. normal and seizure events). However, healthy subjects were recorded both with eyes opened and closed. For the sake of comparison, we removed muscular and ocular artifacts using the algorithm filtration discussed in [37]. Each set contained 160 recorded scalp EEG signals from 20 channels corresponding to the electrodes placed on the head according to the International 10-20 System of Electrode Placement Standard. Set A contains 80 normal recordings (i.e., seizure–free), whereas set E has 80 epilepsy recordings (a neurologist examined the EEG data to identify all epileptic events). Recordings, which were performed under video monitoring to mantain careful observations of different seizure stages, were sampled at a frequency of 256 *Hz* with 12–*bit* resolution and 2-*min* durations. All patients underwent clinical examinations by a neurologist. Two segments of a typical original EEG recording are shown in Figure 2, and illustrate normal and seizure episodes. As shown, measured perturbations obscure the observed brain activity. The data was acquired under non–regulated conditions as evidenced by noise (accounting for wakeful background EEG activity), muscle artifacts, and a 60 *Hz* power line interference. Moreover, DB2 contains only normal and seizure events.

During preprocessing, all EEG recordings of either database were digitally band–pass filtered to focus on the 0-40 *Hz* frequency range. EEG data were normalized to the greatest absolute value of each *i*–th EEG signal. That is,
10

### Computed short–time rhythms

**Figure 3:**
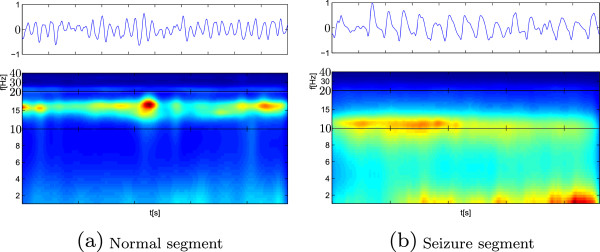
**EEG spectrograms.** Example STFT plots of log–power distributions computed for EEG segments lasting 2.9*s*. **(a)** Normal segment, **(b)** Seizure segment.

Once the signal EEG signal enhancement is accomplished, as described in “Short–time rhythm extraction from enhanced EEG representation”, a set of the subspectral components is extracted from which the rhythms are to be further computed. Figure 3 shows the estimated spectrogram examples of EEG data segments labeled as normal and seizure, respectively. The STFT spectrograms are performed using the Gaussian window of 2.9 *s* length (i.e., 503 samples) and with 40*%* overlapping, as recommended in [28]. As seen from spectrograms, EEG power spectrum of epileptic seizure activity is mainly distributed within the frequency range (0-40) *Hz*, involving all considered rhythm frequency bands.

### Estimated relevance weights of short–time EEG rhythms

The computation of the rhythm relevance measures, or weights, is carried out by estimating their stochastic variability, as described in Algorithm 1.


**Figure 4:**
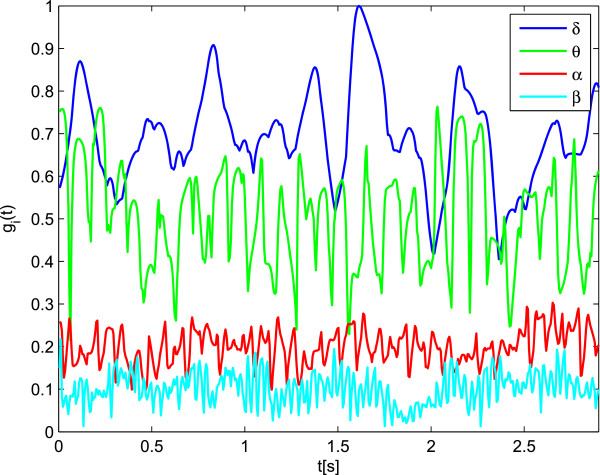
**Estimated rhythm relevance.** Contribution of each EEG rhythm over time computed for DB1, in terms of measured variability as weight of relevance.

For Problem I, Figure 4 shows the estimated time–variant stochastic rhythm weights, ***g***_*i*_(*t*), that are computed within 2.9 *s* EEG segments for both considered databases. As seen, the ***δ*** rhythm represents the greatest relevance weight, exceeding those of the other rhythms. The other rhythms, ranked in descending weights order are: ***θ***, ***α***, and ***β***.

### Performed classification based on short–time rhythms

In order to validate the proposed training methodology, the accuracy of the SVM classifier was investigated, by using a conventional cross–validation procedure which tested each database 10 times.

The commonly used cross-validation procedure performs 10 repetitions where 70*%* of the data is used for SVM training while the remaining 30*%* is used for testing. This strategy provides better parameter estimation because the variance of the resulting estimate is reduced as the number of repetitions is increased. The values of *c* and *ς* are iteratively optimized, so that both parameters change after each test. Once the SVM training concludes, parameter values maximizing the estimator accuracy are used for testing.

This strategy provides an estimation for the model reliability of the model by computing the variability of the results through the 10 repetitions. The performance parameters are accuracy, sensitivity, and specificity, respectively defined as:
111213

where *N*_*c*_ is the number of correctly classified patterns, *N*_*T*_ is the total number of patterns used to feed the classifier, *N*_*TP*_ is the number of true positives (i.e., accurately classified objective classes), *N*_*FN*_ is the number of false negatives (i.e., objective classes classified as reference classes), *N*_*TN*_ is the number of true negatives, and *N*_*FP*_ is the number of false positives.

**Figure 5:**
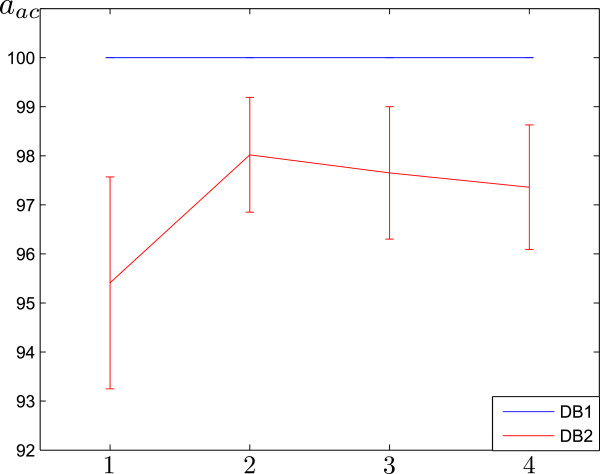
**Performed classifier accuracy.** A–E bi–class problem for both databases, blue line DB1, red line DB2. Classification accuracy was computed when individually adding their weights ranked by their decreasing relevance. As labeled on the horizontal axis: 1: *δ*, 2: *δ*+*θ*, 3: *δ*+*θ*+*α*, 4: *δ*+*θ*+*α*+*β*.

EEG segments were categorized using the support-vector-machines-based with the RBF kernel. To extend the basic binary capabilities of the SVM classifier to a multi–class tool, we employed a one-against-all algorithm to categorize a given input EEG segment among the three studied classes (i.e., normal, interictal and ictal). Figure 5 shows the estimated accuracy computed when individually adding weights of short–time rhythms of the bi–class problem by decreasing relevance. Estimated weights can be grouped according to the following four training scenarios (indicated on the horizontal axis): 1) ***δ*** rhythm, 2) →***δ***+***θ***, 3) →***δ***+***θ***+***α***, and 4) →***δ***+***θ***+***α***+***β***. When validation was carried out over both databases, it is clear that low frequency band rhythms provide significant contributions. The starting contribution of the ***δ*** rhythm is high, and is the greatest when considering both ***δ***+***θ***. However, classifier performances diminished when adding higher low band rhythms.

The scenario 2, has the highest accuracy. The computed average classification measures and their respective standard deviations are presented in Table 1. For DB1, the best obtained classifier accuracy reaches the highest values (*a*_*ac*_=100*%*, *a*_*se*_=100*%*, *a*_*sp*_=100*%*). Likewise, the same behavior was observed when testing was carried out over DB2 after noise reduction. However, for DB2, the achieved classification measures were lower because of residual noise interference and artifact influence. It is worth noting that the higher activity observed in some frequency regions may be due to artifacts or noise. In order to measure the influence of artifacts on the proposed training approach, we remove ocular and muscle artifacts from DB2 using the method discussed in [37]. We computed relevance weights for cases: with and without artifacts. The obtained results are presented in the final row of the Table 1, *DB*2^∗^. Artifact removal provided a slight improvement in the achieved classifier performance, (namely *a*_*ac*_=0.33*%*, *a*_*se*_=0.32*%*, *a*_*sp*_=-0.42). Standard deviations also slightly diminished, indicating the robustness of the proposed approach training.

**Table 1:** **SVM classifier performance results for bi–class problem I**

***DB***	***a***_***ac***_ [ ***%*** ]	***a***_***se***_ [ ***%*** ]	***a***_***sp***_ [ ***%*** ]
*DB1*	**100**	**100**	**100**
*DB2*	98.02±2.16	97.79±1.78	99.31±1.68
*DB2* ^∗^	98.35±1.27	98.11±1.45	98.89±1.47

For classification problems II and III, testing was carried out using scenario 2 (i.e., ***δ***+***θ***) only for DB1, because DB2 was not properly labeled (neither for Problem III). As shown in Table 2, the STFT shows a high classifier performance achieving 100*%* accuracy. For the classification problem III, the performed accuracy measurements are also presented in Table 2 and show high sensitivity and specificity scores. The former is very important because there is a need to significantly reduce the percentage of incorrectly classified epileptic seizures.

**Table 2:** **SVM classifier performance results for the classification problems II and III**

Set	***a***_***ac***_ [ ***%*** ]	***a***_***se***_ [ ***%*** ]	***a***_***sp***_ [ ***%*** ]
A	**100**	**100**	**100**
D		**100**	**100**
E		**100**	**100**
A	96.58±3.09	96.18±4.32	99.38±1.12
B		96.56±2.19	99.16±0.96
C		94.21±4.65	98.32±1.21
D		90.86±4.16	98.54±2.25
E		99.12±1.06	99.13±0.96

It is worth noting that most works in literature on the classification of ictal and interictal activities do not provide validation tests of algorithms based on data collected from different individuals. To develop a blinded validation study, we used the DB2 database, which contains the necessary information on 40 patients. We combined the patient information with their corresponding recording labels to carry out the support-vector-machine classifier cross–validation scheme for the bi–class normal/seizure detection problem. As shown in Table 3, the average accuracy of the patient classification is above 90*%* (the detection accuracy of the normal class is *a*_*ac*_=97.19*%*, while the accuracy of the pathological class is 98.33*%*), with high values of specificity (97.95*%*) and sensibility (97.59*%*). However, the proposed algorithm exhibited poor accuracy for two patients (*#*2 normal, and *#*11 with epileptic seizures).

**Table 3:** **Results for patients classification**

*Class*	*Patient*	1	2	3	4	5	6	7	8	9	10	
*Normal*	*Error seg.*	0	8	3	0	0	0	0	0	0	2	
	*a*_*ac*_[ *%*]	100	83.3	93.7	100	100	100	100	100	100	95.8	
	*Patient*	11	12	13	14	15	16	17	18	19	20	
	*Error seg.*	2	3	0	4	0	0	4	0	0	1	
	*a*_*ac*_[ *%*]	95.8	93.7	100	91.6	100	100	91.6	100	100	97.9	
*Seizure*	*Patient*	**1**	**2**	**3**	**4**	**5**	**6**	**7**	**8**	**9**	**10**	
	*Error seg.*	0	0	0	0	0	0	0	3	0	0	
	*a*_*ac*_[ *%*]	100	100	100	100	100	100	100	93.75	100	100	
	*Patient*	**11**	**12**	**13**	**14**	**15**	**16**	**17**	**18**	**19**	**20**	
	*Error seg.*	6	0	0	0	0	0	0	4	0	3	
	*a*_*ac*_[ *%*]	87.5	100	100	100	100	100	100	91.6	100	93.7	

### Relevance rhythm diagrams in neural activity monitoring

If the solution in the multivariate decomposition of Eq. (4) implies the separated computation of each *k* class matrix set, {***A***^(*k*)^}, each considered class (normal, interictal and seizure) can be reconstructed using Eq. (3). Accordingly, the relevance measure can be calculated by Eq. (6) for each class, {*γ*^(*k*)^}. Table 4 shows the relevance weights computed for bi–class problem I, which were normalized to the maximum value for comparison. In general, the greater the weight, the more relevant the respective short–time rhythm; as such, ***α*** provided the greatest contribution because this rhythm achieved the largest weight for the normal labeled observations (subset A). Similarly, ***δ*** and ***θ*** rhythms are also important, while the ***β*** waveform provided the lowest contribution. For the reconstruction of the seizure state, the rhythm exhibiting the greatest weight was ***δ***, whose increased activity may be an indicator of focal epilepsy in the temporal region of the brain [38]. The second greatest weight was observed in the ***θ*** waveform, which is also commonly associated with epilepsy. The modest values observed in the estimated ***β*** relevance weights indicate its low contributions.

**Table 4:** **Estimated relevance weights per class from considered short–time rhythms for both underlying data bases**

DB		***δ***	***θ***	***α***	***β***
**DB1**	*Seizure*	1-0.03	0.93±0.06	0.52±0.06	0.26±0.09
	*Normal*	0.71±0.05	0.82±0.06	1-0.03	0.42±0.12
	*Diff*	+	+	-	-
**DB2**	*Seizure*	1-0.07	0.85±0.08	0.58±0.12	0.25±0.09
	*Normal*	0.69±0.05	0.78±0.02	1-0.07	0.36±0.12
	*Diff*	+	+	-	-

**Figure 6:**
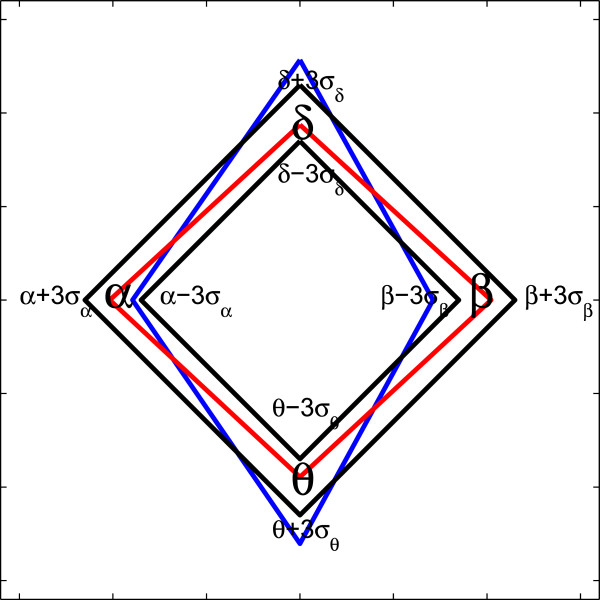
**Relevance rhythm diagram.**

Assuming that the normal brain state represents baseline neural activity, the “*Diff*” row in Table 4 indicates a positive or negative variation of reconstructed rhythm relevance weights from baseline for an epileptic seizure activity. As shown, both ***δ*** and ***θ*** short–term rhythms increased brain activity(i.e., “ + ”), while ***α*** and ***β*** rhythms reduced brain activity (i.e., " -"). This indicates that the patterns were substantially affected by the clinical condition of the patient. Variations of each rhythm energy distribution from one neural brain state to another can be illustrated by introducing the relevance rhythm diagram (RRD), which is an orthorhombic–shaped strip of uniform width, computed in such a way that relevance weights of a given referenced neural state must be confined within a narrow region (typically, within a 3*σ* width of corresponding rhythm values). Figure 6 shows an example of an RRD for normal and ictal signals. Here, the signals of the reference class are circumscribed (red), while the signals of the compared class present variations in the energy of its rhythms (blue). In this case, the actual signal increased values in the delta and theta bands, as compared with the reference normal signal.

**Figure 7:**
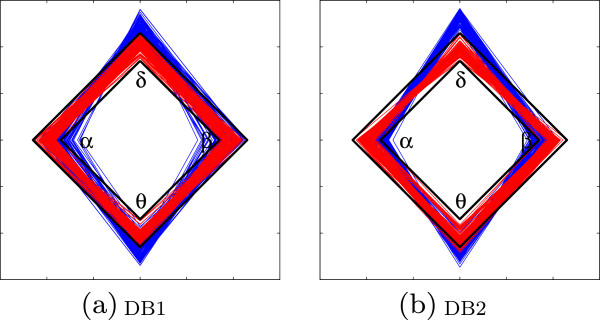
**Computed relevance rhythm diagrams.** Obtained A ^∗^–E RRD for Problem I. Normal, baseline events (A) are depicted with red lines while seizure–related events (E) are shown with blue lines. **(a)** DB1, **(b)** DB2.

To better interpret the assessed weights for both neural brain states, the rhythm measurement location was intentionally changed. To draw an RRD, relevance weights were estimated for a set of 23.6 *s* single–channel EEG segments. For Problem I (A^∗^–E), the corresponding RRD is shown in Figure 7(a) for DB1 and in Figure 7(b) for DB2, respectively. The notation ^∗^ indicates the referenced neural state. In both cases, normal brain activity related weights were mostly within a 3*σ* width for all considered rhythms. Both fixed moments increased the clinical interpretation of monitored EEG data.

For epileptic events, the shape of the seizure–related diamond becomes strained, i.e., the vertical axis is longer while the horizontal axis tends to be shorter. The same situation was observed for the labeled DB2 set; however, a few overlapping values were presented in ***α*** and ***β*** corners. Low rhythm bands (i.e., ***δ*** and ***θ***) increased their contribution to overall rhythms, while high rhythm bands (i.e., ***α*** and ***β***) presented diminished activity.

**Figure 8:**
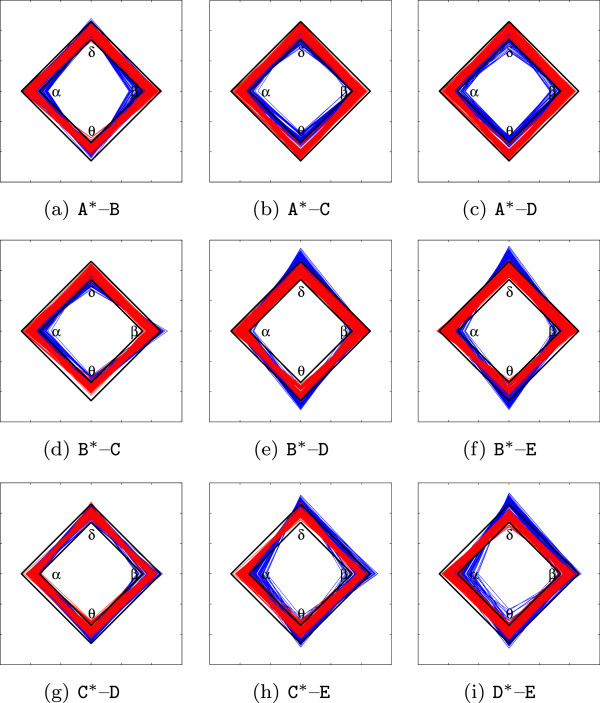
**RRD examples for Problems II and III.** Diagrams are calculated for the referenced classes noted with asterisks. **(a)** A ^∗^–B, **(b)** A ^∗^–C, **(c)** A ^∗^–D, **(d)** B ^∗^–C, **(e)** B ^∗^–D, **(f)** B ^∗^–E, **(g)** C ^∗^–D, **(h)** C ^∗^–E, **(i)** D ^∗^–E.

Likewise, the accomplished RRD representations of Problems II and III are shown in Figure 8. For the sake of simplicity, the top row holds RRD, for which subset A is the referenced neural activity, middle row holds the B-referenced RRD, and the bottom row has both remaining C-referenced and D-referenced RRD representations. In all cases, the referenced class is noted with asterisk. As seen in the top row, all rhythm weights estimated for classes C and D exhibit different behavior if compared to normal brain activity (subset A^∗^), in which rhythm weights tend to be higher. When compared to class B, however, only the high normal class rhythms (***α*** and ***β***) increase their contribution. That is, if the referenced class becomes the B class, the obtained RRD shows rhythm contributions that are different from all compared classes. Regarding the epileptic seizure free zones, subset C mostly tends to be confused with D, as already discussed in [6].

## Discussion

This work proposes a methodology to quantitatively evaluate every short–time rhythm contribution to neuronal activity for the purpose of discriminating between normal and interictal/ictal activities. The discussed methodology hypothesizes that by providing appropriate and relevant measures of time–variant rhythm waveforms, one can differentiate different neuronal activities. There are two reasons this methodology was used for the diagnosis of epilepsy: clinical interpretability (the rhythm concept is well–known) and easy implementation (feature extractions and classifier processes are based on simple inference and do not require complex calibrations). The methodology benefits from deeper study of the signals over time. While the obtained preliminary results are encouraging, additional aspects should be further consider:

- The first aspect concerns the EEG enhancement method, from which short–time rhythms are extracted. The stochastic feature vector estimated from STFT is used because it is able to reveal non-stationary dynamics of EEG signals. Although a large amount of filterbank-based features have been proposed to characterize sub–band rhythms, the LFCC parameters are chosen as the feature vectors because of their simple, but effective, combination of frequency and magnitude from the short-term power spectrum of EEG signals. LFCC parameters can be accurately calibrated to each rhythm frequency band. However, this stochastic relevance analysis strategy should be tested on nonlinear methods of feature extraction (e.g., entropies, fractal dimension, and recurrence quantification) that have been found to be effective for more accurate diagnose of epilepsy [39].
- Moreover, the classifier performance of neural activities is similar to values reported in the literature for other commonly used enhanced representation approaches (e.g., WT and EMD), as shown in Table 5. However, because the purpose of the discussed training methodology is the brain activity monitoring, only a few strategies are suitable for a concrete time–frequency EEG decomposition approach. The criteria used for the STFT window may greatly influence classifier performance and the estimation of the RRD. Another shortcoming of the STFT is its computational burden that over–exceeds the WT by one order of magnitude. However, matching each WT level with the appropriate rhythm frequency sub–band depends at least, on proper sampling frequencies; this deficiency is discussed in detail in [10]. The possible disadvantages of EMD may be related to low–frequency mixing issues and the non–differentiability of the phase function that may be observed with a strong artifacts.

**Table 5:** **Classification accuracy (%) for the detection of epileptic seizures as reported by the discussed stochastic relevance method and by other recent works**

Authors	Features/Classifier	Subset	***a***_***ac***_ [ ***%*** ]
[28]	TFR-2DPCA/*k*-nn	A,E	**100**
[42]	*t-f* analysis/RNN	A,E	99.60
[43]	WT/PNN	A,E	99.99
[44]	PCA FFT/AIRS	A,E	**100**
[41]	CC+PSD/voting of classifiers	A,E	**100**
[7]	*t-f* analysis/ANN	A,E	**100**
*This work*	short–time rhythms/*k*-nn	A,E	99.50
*This work*	short–time rhythms/SVM	A,E	**100**
[19]	PCA-RBF/ANN	A,D,E	96.60
[40]	EV/MLP NN	A,D,E	97.50
[45]	PSD+CLZ/SVMA	A,D,E	98.72
[28]	TFR-2DPCA/*k*-nn	A,D,E	98.80
[7]	*t-f* analysis/ANN	A,D,E	100
*This work*	short–time rhythms/*k*-nn	A,D,E	98.12
*This work*	short–timerhythms/SVM	A,D,E	**100**
[7]	*t-f* analysis/ANN	A,B,C,D,E	89.00
[28]	TFR-2DPCA/*k*-nn	A,B,C,D,E	94.40
[6]	(WT + eigenvectors)/SVM	A,B,C,D,E	99.20
*This work*	short–time rhythms/*k*-nn	A,B,C,D,E	95.78
*This work*	short–time rhythms/SVM	A,B,C,D,E	96.58

- The next consideration is related to the stochastic relevance analysis of time–evolving rhythm waveforms, which was proven to maintain a discriminant capability for neural activity detection and monitoring. As seen in Table 4, estimated rhythm weights do not significantly change over different databases, indicating their robustness toward noisy acquired EEG data. To the best of our knowledge, there are no known approaches that measure the concrete contribution of each physiological rhythm to discriminate neural states (normal, interictal, and ictal). The proposed stochastic relevance analysis may help medical specialists concretely determine the contribution of physiological rhythms for the identification and monitoring of brain neural activity during long time periods.
- There are two main reasons for choosing the proposed diagnosis strategy for epilepsy: clinical interpretability (clinicians are familiar with the rhythm concept) and easy implementation (feature extraction and classifier processes are based on simple inference and do not require complex calibration). The methodology benefits from a deeper study of the signals over time.
- Based on the estimated rhythm relevance weights, classifier performance is carried out, as shown from Table 1. For Problem I, the highest classifier accuracy is obtained (namely, *a*_*ac*_=100*%*, *a*_*se*_=100*%a*_*sp*_=100*%*) for DB1. For the noisy DB2, high classifier performance is also obtained (*a*_*ac*_=98.02*%*, *a*_*se*_=97.79*%*, *a*_*se*_=99.31). However, because practicing neurologists have difficulty in differentiating between interictal and healthy EEG recordings, solving Problem II (instead of Problem I) is more relevant to the medical community [40]. As shown in Table 2, the proposed training methodology achieves 100*%* accuracy, making it more suitable for the implementation of EEG monitoring systems.
- The accuracy values of the proposed training approach and other recent approaches are compared in Table 5, using DB1 and problems (I,II, and III). Although this comparison may not be completely fair due to different details on the testing procedures (there is a wide dispersion in the choice of the analysis window; see [4–8]), it seems to be the best possible option. For bi–class and three–class problems, our obtained SVM classifier offers the best accuracy. It is worth noting that rhythm waveforms, have direct clinical interpretability. Furthermore, our classification approach still produces high accuracy when employing a simple *k*–nearest neighbor classifier, which simplifies the training design complexity. The optimal value was *k*=3, which is close to the *k* values in similar works [7, 41].– In addition to the conventional quantitative discrimination analysis (based on classifier performance), another practical consideration uses the relevance evaluation of short–time EEG rhythms to the qualitative identification of brain neuronal activity. However, few studies have determined proper physiological EEG rhythm parameters for use as classifier inputs. The discussed methodology for the relevance evaluation of EEG rhythm waveforms may provide a qualitative identification of epileptic seizures. From the assessed stochastic relevance analysis, the introduced relevance rhythm diagrams permit the qualification of the contribution of neural dynamics for each patient’s condition, enabling improved clinical interpretations of obtained results (Figures 7 and 8).
- Therefore, as particular cases of piecewise linear methods, short–time rhythms can be highly relevant and similarly effective than nonlinear methods for the characterization of neuronal dynamics, in terms of classifier performance. Because of their straightforward interpretation, this characterization may yield valuable diagnostic information. However, there are no standard frequency ranges for determining these different bands [41]. Certain variability should be considered between subjects.
- The average computational time for extracting the feature vector from a single EEG segment was 0.9293*s*. To reduce computational burden, the use of feature extraction based on faster filterbank decompositions (particularly, wavelets) should be strongly considered. This approach was suggested in [46] in which a bi-class problem is addressed where the computation of the feature vector over a single EEG segment was nearly 100 times faster.

## Conclusions

This work proposes a methodology to quantitatively evaluate each waveform contribution to the neuronal activity related to either normal or epileptic seizure states. A relevance evaluation is based on a time–evolving, latent variable decomposition of electroencephalogram signals. The discussed methodology is simple and can interpret the assessed feature set. The methodology is based on the hypothesis that using relevance–based analysis over enhanced representations of EEG signals permits measurements of rhythm contributions in each clinical case. The proposed methodology uses STFT as a decomposition method to extract sufficient information from the short–time EEG rhythms. In turn, measured rhythm relevance weights provide high classifier performances in terms of distinguishing between brain neural activities.

These results can be used in future studies focusing on finding alternative methods for monitoring and diagnosing epileptic seizures using less costly and noninvasive equipment. The introduced rhythm relevance weights have the added benefit of providing easier clinical interpretations, we additionally introduced the relevance rhythm diagram, which provides a qualitatively measure of the rhythm contribution to the neural activity. This may be used during EEG data monitoring. The proposed methodology of stochastic relevance analysis can be translated to other monitoring applications involving time–variant biomedical data.

Future areas of research include the application of the discussed methodology to analyze other brain activities and to determine the feasibility of seizure prediction. More elaborate and higher accuracy EEG analysis techniques (e.g., neural activity mapping) can also be considered for the diagnosis of epilepsy.
